# Unexplored Challenges of Minoritized Microbiologists in Academia

**DOI:** 10.1128/aem.01514-22

**Published:** 2022-11-17

**Authors:** Alfredo G. Torres

**Affiliations:** a University of Texas Medical Branch, Department of Microbiology and Immunology, Galveston, Texas, USA; Michigan State University

**Keywords:** diversity, equity, inclusion, minoritized microbiologist, underserved microbiologist

## Abstract

The scientific community is making significant efforts to be inclusive and to promote diversity and equity. The microbial sciences are not the exception, and organizations, such as the American Society for Microbiology (ASM), are implementing strategic plans to advance these initiatives. However, one unexplored topic is whether the recruitment of minoritized microbiologists should use tailored programs for the success of trainees and faculty. Some challenges and opportunities are presented for consideration while developing recruitment, retention, and advancement programs in the microbial sciences.

## TEXT

Microbiology departments across the United States, as parts of major universities, colleges, and institutes, are joining a growing national movement to diversify their institutions and to advance their inclusion and equity missions. However, one major, common concern by search committees that recruit students and faculty is that is exceedingly difficult to find qualified individuals, particularly those that belong to minoritized groups (e.g., Black, Hispanic/Latinx, Indigenous [American Indians]). Although this concern represents a common bias permeating academia, it does not reflect the available qualified candidates that can occupy those positions, and search committees need to consider the challenges of this group of applicants while recruiting them. Schools continue to develop strategic plans to recruit these microbiologists, but they do not consider that trainees and/or faculty candidates belonging to these ethnic and racial groups might require different strategies to be incorporated in their scientific/academic communities or that there is a need to create a change in the academic culture that can support their careers and their advancement in the microbial sciences (1, 2). Here are some concepts that might not have been previously evaluated as parts of the decision-making strategies established by microbiology departments, centers, institutes, or associations across the nation.

## BLACK MICROBIOLOGISTS HAVE A LONG TRADITION OF CREATING SYSTEMS/NETWORKS THAT SUPPORT THE ADVANCEMENT OF THEIR CAREERS

It is well-documented that Black scientists are historically excluded in microbial education, training, and research (3, 4); however, there is a long tradition of exceptional Black microbiologists contributing to various aspects of the microbial sciences, as depicted in the 100 Inspiring Black Scientists in America report (5). Further efforts to recognize the contributions of Black microbiologists through history, emphasizing their courage and determination while facing racial discrimination, have been made by microbiology scientists while celebrating the International Microorganism Day (6) and by the American Society of Microbiology (ASM) while celebrating their Black Clinical Microbiologists (7). A feature of the current members of the Black scientific community is their remarkable resilience and their ability to support each other through networks and organizations, regardless of the institution to which they belong, that has helped them to create a sense of community. One of the best examples of the network system supporting these scientists was the establishment of the Black Microbiologists Association (BMA) in 2020 (8), which was intended to reduce the underrepresentation of Black scientists in microbiology. BMA has organized two #BlackInMicro weeks to highlight the research of Black microbiologists and to connect and create a sense of community. These events are ideal platforms for the recruitment of trainees and faculty candidates to the different universities, societies, and nonacademic institutions that are trying to diversify their scientific communities or connect their constituents to a network that will support their careers.

ASM has recognized the value of the Black community of microbiologists by having the only Black President of the Society on Clifford Houston of the University of Texas Medical Branch, who was a microbiologist who advanced the educational mission of ASM and founded the Annual Biomedical Research Conference for Minority Students (ABRCMS). Unfortunately, the recent ASM Diversity, Equity and Inclusion Task Force Report (9) showed that Black microbiologists make up only 5% of ASM’s members, and other data suggest that they represent less than 3% of all microbiologists in the United States (10). Further efforts are needed to increase and advance this community of scientists. In fact, Black microbiologists still are facing systemic racism and/or biases as roadblocks for their advancement (11). Therefore, we must use discussions about diversity and inclusion, occurring during the “racial awakening” currently happening in the United States, to work together with recruiters, academic leaders, and decision-making individuals to guarantee that the next generation of microbiologists is better represented in the microbial sciences.

## HISPANIC, LATINX, LATIN AMERICAN, PUERTO RICAN: THE NEED TO UNIFY HISPANIC MICROBIOLOGISTS TO SUPPORT THEIR CAREERS

Hispanics are the fastest growing minoritized group in the United States, but they remain a significantly underrepresented group in the microbial sciences, comprising 6% of the total workforce of microbiologists in the United States (10, 12, 13). Because Hispanics have many commonalities, they are routinely treated as a homogeneous group in science and research (14). As such, the perception that this ethnic group has made significant advances in the numbers of trainees and faculty members joining different academic institutions is due to a broad definition that encompasses individuals of Hispanic/Latino descent (15). At least three large groups can be included in the definition of Hispanic: Latinos/Hispanics born and trained in the United States, Hispanics migrating from Latin America and Spain, and Puerto Ricans that trained on the island or the mainland and have either remained there or migrated from the island. Each of these subgroups has its own strengths and experiences different challenges in career advancement.

In general terms, the first subgroup consists of individuals that were born and educated in the United States, and microbiologists from this group are mainly distributed in Hispanic-serving institutions (16) or in states (e.g., California, Texas, Illinois, New York, and Florida) with a large representation of Hispanic students in science, technology, engineering, and mathematics (STEM) (17). These individuals are good recruitment ambassadors for their institutions. A second subgroup includes faculty members and some trainees who did most of their training in Latin America or Spain and were recruited as academicians by United States institutions. These microbiologists have, in many cases, strong networks within their countries of origin, and they have the opportunity to recruit trainees from those countries to their laboratories (18–20).

Finally, a third subgroup consists of microbiologists with a strong link to Puerto Rico, either because they trained there and are part of the academia of the island or because they are Puerto Rican trainees or faculty members on the mainland. These microbiologists have a long tradition of training excellent students who are frequently recruited by different institutions to be part of their graduate programs, but they are less often recruited to become faculty members. Interestingly, Puerto Rico is the place that has the most ASM branches housed at its academic institutions, serving as a forum for microbiology trainees to collaborate and to organize local scientific meetings. Further, the Puerto Rican microbiologists have established support networks in the form of “Ciencia Puerto Rico” (https://www.cienciapr.org/) and networks for professional development as part of the Puerto Rico Society of Microbiologists (https://www.micropr.org/). However, the economic challenges of the island lead many microbiologists to migrate to the mainland and hinder their direct participation in these networks.

The classification of subgroups of Hispanic microbiologists was made to highlight both the wide variety of individuals that encompass this nonhomogenous ethnic group and the fact that many of these microbiologists do not have many commonalities, which complicates the establishment of collaborations and the creation of support networks. It is evident that Hispanic microbiologists continue to be the fastest growing group in the Microbial Sciences, but without creating adequate support networks, it might be difficult to increase recruitment by different institutions. ASM needs to have a Hispanic President who can serve as a unifier of the Hispanic microbiologists in the United States.

## INDIGENOUS MICROBIOLOGISTS: A CALL TO SUPPORT THIS MINORITIZED GROUP

Compared with other underserved groups in academia, indigenous microbiologists number significantly fewer, and those involved in academic positions are often focused on education. Due to the limited number of American Indian microbiologists, these individuals need to join other established networks that understand their needs and can support their advancement and success in the microbial sciences. Joining other networks is not ideal because the indigenous community must create a network that can promote trainees in microbiological careers and let them advance into academic positions. Further, the need to increase the number of these microbiologists is of particular importance, as many infectious diseases disproportionately affect indigenous people (21), posing significant public health challenges for these communities. Interestingly, in many other fields of science, American Indians have critical mass, but, in the case of microbiology, the number of established Indigenous investigators does not exceed more than 10. Therefore, ASM, as a part of their strategic inclusive diversity with equity, access, and accountability (IDEAA) strategic plan (22), needs to establish clear actions that can empower these microbiologists in support of their professional careers.

## ABRCMS AS A SOURCE OF MINORITIZED TALENT FOR ACADEMIA

Many conferences and scientific meetings are constantly highlighting the nonbiomedical efforts to build a diverse and welcoming community for professionals in various STEM fields. Venues that include basic and/or applied microbiology topics are trying to diversify their panels of speakers and participants; however, sometimes the pool of participants from minoritized groups seems exceedingly small. For 20 years, the Annual Biomedical Research Conference for Minority Students (ABRCMS; https://abrcms.org/), recently renamed the Annual Biomedical Research Conference for Minoritized Scientists, has been the place for minoritized individuals from community colleges as well as undergraduate, graduate, and postbaccalaureate students to display their talents in STEM. This conference also brings together many minoritized faculty members that serve as mentors, judges, speakers, and recruiters of all of the students who attend the conference. ASM has sponsored this conference from the beginning, with the goals being to promote microbiology and other STEM fields and to use this platform to empower the next generation of microbiologists. Academic institutions that are trying to diversify their research programs or faculty pools at large and cannot find sufficient candidates for their graduate programs should consider attending this conference and developing long term strategic plans to support these minoritized scientists in training, with the goal being to recruit those who demonstrate excellence in their research and can eventually occupy faculty positions at academic institutions.

## CONCLUDING REMARKS

This commentary is not intended to be a comprehensive review of all of the challenges and opportunities faced by minoritized microbiologists. Instead, this commentary is a series of personal observations that could be useful for academic institutions and microbial societies that are trying to diversify their microbiology programs by having a better understanding of the existing networks supporting these individuals and the potential mechanisms that can be established to support the recruitment, retention, and advancement of minoritized microbiologists. It is evident that recruiters of different institutions need to consider establishing collaborations with some of these networks so that equity in the pool of applicants can be created (23). Here are some activities that can be established at the institutions or in partnership with ASM to increase the recruitment of these individuals. First, the establishment of faculty coalitions within the institution or with different institutions in the same state or region that can support the recruitment, onboarding, and mentoring of minoritized microbiologists is critical. Second, to prevent the isolation of these recruits, institutions should consider the cluster-hiring of individuals, in which supportive and collaborative networks are created between these subgroups in the institution or in the regional/state institutional setting to increase the sense of community. Third, it is critical for academic institutions to create a strategic plan that matters (24) as a part of their diversity, equity, and inclusion (DEI) efforts in order to define how recruited minoritized microbiologists can find the optimal conditions for their success and advancement in an academic environment that fully supports them. For example, they can create partnerships with ASM or with the National Institutes of Health (NIH) MOSAIC (Maximizing Opportunities for Scientific and Academic Independent Careers) award program, which is intended to promote the independent careers of diverse faculty in research-intensive institutions (https://www.nigms.nih.gov/training/careerdev/Pages/MOSAIC.aspx). Fourth, representing the most vital component of any institutional DEI plan, which is commonly ignored, is that when minoritized microbiologists become successful, they will be heavily recruited by other programs. Therefore, the home institutions that have created a more inclusive culture and environment for trainees and faculty will be more successful in retaining their talented minoritized microbiologists. Finally, it is always important to emphasize that recruiting minoritized individuals just to fulfill a quota established by the institution, without a well-designed DEI strategic plan, is a disservice to the microbiological community at large and to those minoritized microbiologists who deserve more opportunities to display their talents.
